# American Ginseng (*Panax quinquefolium* L.) as a Source of Bioactive Phytochemicals with Pro-Health Properties

**DOI:** 10.3390/nu11051041

**Published:** 2019-05-09

**Authors:** Daria Szczuka, Adriana Nowak, Małgorzata Zakłos-Szyda, Ewa Kochan, Grażyna Szymańska, Ilona Motyl, Janusz Blasiak

**Affiliations:** 1 Institute of Fermentation Technology and Microbiology, Lodz University of Technology, Wolczanska 171/173, 90-924 Lodz, Poland; ilona.moty@p.lodz.pl; 2 Institute of Technical Biochemistry, Lodz University of Technology, Stefanowskiego 4/10, 90-924 Lodz, Poland; malgorzata.zaklos-szyda@p.lodz.pl; 3 Pharmaceutical Biotechnology Department, Medical University of Lodz, Muszynskiego 1, 90-151 Lodz, Poland; ewa.kochan@umed.lodz.pl (E.K.); grazyna.szymanska@umed.lodz.pl (G.S.); 4 Department of Molecular Genetics, Faculty of Biology and Environmental Protection, University of Lodz, Pomorska 141/143, 90-236 Lodz, Poland; janusz.blasiak@biol.uni.lodz.pl

**Keywords:** *Panax quinquefolium* L., ginsenosides, anti-cancer activity, anti-diabetic potential, antimicrobial effect

## Abstract

*Panax quinquefolium* L. (American Ginseng, AG) is an herb characteristic for regions of North America and Asia. Due to its beneficial properties it has been extensively investigated for decades. Nowadays, it is one of the most commonly applied medical herbs worldwide. Active compounds of AG are ginsenosides, saponins of the glycosides group that are abundant in roots, leaves, stem, and fruits of the plant. Ginsenosides are suggested to be primarily responsible for health-beneficial effects of AG. AG acts on the nervous system; it was reported to improve the cognitive function in a mouse model of Alzheimer’s disease, display anxiolytic activity, and neuroprotective effects against neuronal damage resulting from ischemic stroke in animals, demonstrate anxiolytic activity, and induce neuroprotective effects against neuronal damage in ischemic stroke in animals. Administration of AG leads to inhibition of hypertrophy in heart failure by regulation of reactive oxygen species (ROS) in mice as well as depletion of cardiac contractile function in rats. It also has an anti-diabetic and anti-obesity potential as it increases insulin sensitivity and inhibits formation of adipose tissue. AG displays anti-cancer effect by induction of apoptosis of cancer cells and reducing local inflammation. It exerts antimicrobial effects against several pathogenic strains of bacteria. Therefore, AG presents a high potential to induce beneficial health effects in humans and should be further explored to formulate precise nutritional recommendations, as well as to assess its value in prevention and therapy of some disorders, including cancer.

## 1. Introduction

For centuries, phytochemicals have played a significant role in human health protection and treatment of many diseases. These plant-derived substances are reported to display anti-cancer, antimicrobial and anti-diabetic activity [1]. They were also reported to diminish the risk of several disorders such as cardiovascular and neurodegenerative diseases [2]. 

*Panax quinquefolium* L. (American Ginseng, AG) is an example of a plant rich in bioactive phytochemicals. Its active compounds—ginsenosides—have been documented to exert a wide range of different biological activities resulting in hypoglycemic, anti-inflammatory, cardio protective, and anti-tumor effects [3,4]. A therapeutic potential of AG in chronic obstructive pulmonary disease has been also suggested [5]. It can also act as an agent diminishing unpleasant menopause symptoms [6].

By now, reviews of AG focused mostly on its chemical analysis and ecological aspects of its use and health-related activities were mainly limited to nervous and cardiovascular systems [7,8,9]. Recently, some reviews addressing molecular targets in pharmacological activities of AG components were published [10,11,12]. 

The review updates information on general properties of AG and focuses on its anti-diabetic, anti-obesity, anti-cancer, anti-aging, and antimicrobial activities. Special attention is paid to the metabolism of ginsenosides by intestinal microbiota and the action of AG in nervous, cardiovascular, and gastrointestinal systems is briefly discussed.

## 2. American Ginseng: Cultivation, Characteristics, and Applications

The *Panax* genus plays an important role among natural compounds applied in human healthcare. Within its 11 species, the three most commonly used are *Panax ginseng* (Asian ginseng), *Panax notoginseng* and *Panax quinquefolium* (American Ginseng). All three species have received significant attention due to their profitable features and have been implemented in healthcare products and food additives all over the world [13,14,15]. North Asia countries, namely eastern regions of China, Japan, and Korea are abundant in Asian ginseng. Notoginseng is an herbal pharmaceutical of Chinese origin and is cultivated mainly in China [16,17].

AG as opposed to Asian ginseng and notoginseng is an herb characteristic for regions of North America. It inhabits areas from Quebec to Manitoba in Canada to Georgia, Louisiana, Arkansas, and Oklahoma in the United States [18]. The greatest area of AG cultivation is located in Wisconsin [19]. Since 1980s this species of ginseng is also cultivated in China [20]. 

AG represents perennial, forest herbs [21]. Individual leaves vary in shape from lance to oblong ones. In the summer small flowers of white colour appear. They are located on a simple umbel within the major leaf axis. The appearance of flowers is followed by berry-like red fruits that contain up to three seeds. Ginseng roots are variably branched and have fleshy white colour. Sometimes, when the plant grows older, it displays an auxiliary root that can be used as a “spare” in the event of damage of the major root [22].

AG is fertilized by generalist insects such as small Halictid bees and has a mixed-mating breeding system. Its process of reproduction is based exclusively on seeds and occurs after a pre-reproductive period that lasts about 3 years. First, green fruits appear in July and August and they accomplish maturity and redness between August and November [18]. Harvesting of roots is possible after 4 years from seeding. However, it should be underlined that cultivated roots are usually harvested after 3–4 years of growth, while wildly grown ginseng is usually harvested at the 8th year of growth or later [23,24].

Thermally processed ginseng roots can be classified into 2 types: fresh (red) and dried (white) [25]. Roots that are subjected to sun dehydration are called white ginseng. Red ginseng is the one after thermal processing with application of steam and high temperature that result in damage to enzymes that cleave active compounds [26]. This procedure stabilizes ginseng without changes in its activity, which is the same as the activity of fresh root. Such thermal processing of ginseng roots also prolongs their shelf life, so dried roots are preferable in natural Asian medicine. Both forms of AG have similar chemical compositions and pharmacological properties [27].

Ginseng medical application in Eastern Europe dates back 2000 years. However, the name of the genus, *Panax* (*pan* from Greek means “all”, *anox* means “treat” which altogether can be understood as “treats all diseases”) was given to it in the first half of the 19th century by Russian scientist Carl Meyer [28].

Varieties of products containing AG are currently available on the market, starting from powders and pellets, ending up on teas. There are roots of ginseng in a form of dried shredded slices and extracts of this plant. Ginseng dried flowers or flakes are also available [29]. In many countries ginseng is being implemented in hair conditioners, shower gels, lotions, and shampoos. Its roots and rhizomes are used as diet supplements, drugs, and finally as food. In the USA there are candies and drinks containing AG extract; in Korea, soups and salads containing ginseng are common, while in China ginseng extract is added to alcoholic beverages [27,28,29,30].

## 3. Bioactive Phytochemicals of American Ginseng

Bioactive compounds responsible for variety of beneficial activities of AG in humans are called ginsenosides or panaxosides. From the chemical point of view, these compounds are glycosides consisting of a non-sugar part—an aglycone and a sugar chain or chains. In the chemical structure of ginsenosides, three types of aglycones can be distinguished: tetracyclic of dammarane type, pentacyclic of oleanolic acid type and tetracyclic of the ocotillol type.

The sugar part of saponins includes hexoses (glucose, galactose), 6-deoxyhexoses (furanose, rhamnose), pentoses (arabinose, xylose) and uronic acids (glucuronic acid). They are usually in cyclic form and are linked to the aglycone by semi-acetal bonds [31,32].

Ginsenosides are designated “Rx”, where “R” means root, and “x” describes, in alphabetical order, the polarity of the compound according to their mobility on thin layer chromatography plates, from index “a” to “h”. For example, the Ra metabolite is the least polar compound. Most ginsenosides consist of a dammarane skeleton with 17 carbon atoms arranged in four rings. Due to the number of hydroxyl groups these compounds can be classified into two main categories: protopanaxadiol (PPD) and protopanaxatriol (PPT) [33].

Protopanaxadiols are the dammarane-type ginsenosides with sugar moiety at C-3 and/or C-20. Their structures are also characterized by a linear linkage between glycosyl chains and acylation occurring at the 6-OH of the terminal glucose of a three-sugar chain. Within this group the Rb1, Rb2, Rb3, Rc, Rd, Rg3, and Rh2 can be distinguished. Over 30 ginsenosides belonging to PPD type are classified to the Rb group. Protopanaxatriols are dammarane-type ginsenosides and they include ginsenosides Re, Rf, Rg1, Rg2, Rh1, F1, F3 and notoginsenoside R1. However, in the PPT moiety there is a hydroxyl group at C-6 distinguishing them from PPD [34,35]. Other features of PPT structures are at most two glycosyl chains and a linear linkage of saccharide chains [34]. Ginsenosides Rb1, Re, Rd, Rg1 and Rb3 are considered as six major saponins and make up more than 70% of total ginsenoside content in AG [35].

Another constituent is oleanolic acid, which contains a pentacyclic triterpene skeleton [36]. Its derivative is ginsenoside Ro [37]. Last but not least ocotillol that has a five-membered epoxy ring at C-20 needs to be mentioned [38]. An example of ocotillol-type panaxoside isolated from the roots and leaves of AG is pseudoginsenoside F11 (p-F11) [39]. 

Two ginsenosides are considered as major marker compounds that could discriminate *P. ginseng* and AG. These are ginsenoside Rf, present in Asian ginseng and p-F11occurring in AG [40]. It was documented that AG is a source of unsaturated fatty acids, including linolenic acid, which is especially important as its consumption decreases frequency of chronic diseases such as arrhythmia and arthritis [41]. Furthermore, ginseng roots contain polysaccharides. They are made up of a complex chain of monosaccharides rich in l-arabinose, d-galactose, l-rhamnose, d-galacturonic acid, d-glucuronic acid and d-galactosyl residues [42]. Wang et al. isolated a novel neutral polysaccharide from the roots of AG [43]. Monosaccharide composition analysis demonstrated that it consisted of glucose and galactose in a molar ratio 1:1.15. It was observed to hinder inflammation by inhibition of inflammatory-related mediator nitric oxide (NO) and cytokines, including tumor necrosis factor (TNF), interleukin 6 (IL-6), and interleukin 1. This indicates AG potential for application in diseases linked with inflammation, including atherosclerosis [43].

Apart from saponins and polysaccharides AG contains terpenes, phenolic compounds, amino acids, flavonoids, volatile oils, vitamins, and minerals [44,45]. A study conducted by Kochan et al. indicated that the content of triterpene saponins in hairy root cultures of AG can be increased by application of *trans*-anethole as elicitator [46]. It was observed to stimulate production of 9 different ginsenosides: Rb1, Rb2, Rb3, Rc, Rd, Rg1, Rg2, Re, and Rf, among which the Re metabolite was documented with the highest rate of production, 3.9 fold in comparison to untreated root [46].

**Figure 1:**
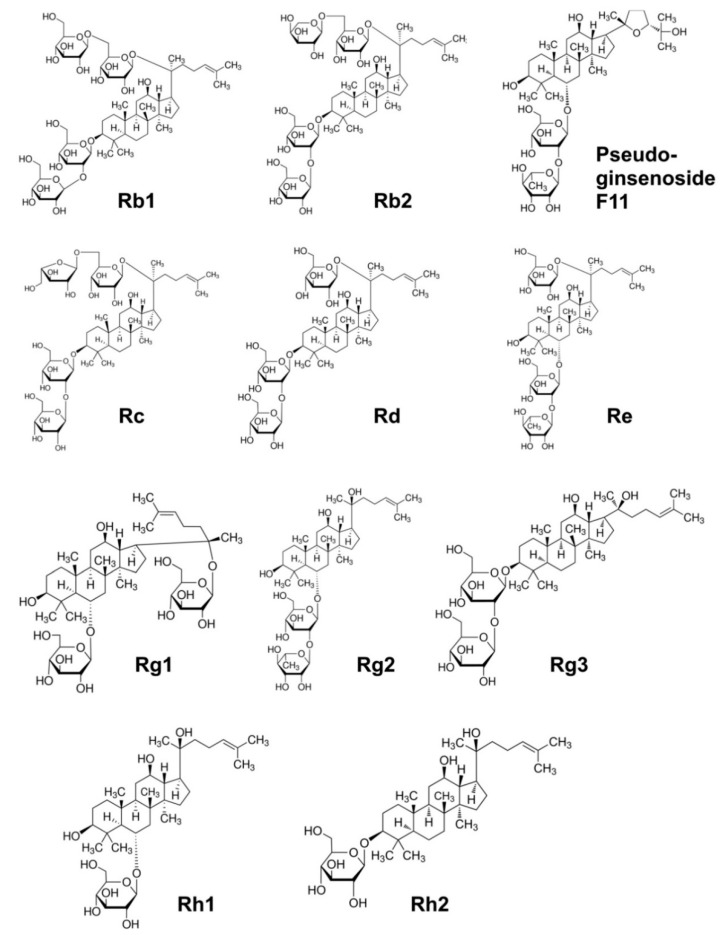
Chemical structure of main ginsenosides present in American Ginseng.

Although all ginseng plants contain either protopanaxadiols (PPD group) or protopanaxatriols (PPT group), their compositions may be different. Asian ginseng contains mainly Rb1, Rb2, and Rg1 ginsenosides [47]. Notoginseng is rich in ginsenosides Rb1, Rd, Rg1 and notoginsenoside R1, while AG in ginsenosides Rb1, Rd and Re [47]. It was also observed that the same species of AG cultivated in different locations display differences in chemical composition, including content of active compounds, and because of that they display different health beneficial activities [48,49]. In addition, many reports confirm that with the age of ginseng plant, the content of saponins increases [23,50,51]. It is also worth mentioning, that although pharmacopoeia raw material of ginseng is root, ginsenosides also occur in other organs of ginseng plant such as: leaves, stems, fruits, and even in small amounts in the seeds [50,52]. Chemical structure of some AG ginsenosides, as well as their pharmacological activities are presented in Figure 1 and Table 1, respectively.

## 4. Pro-Health Effects of American Ginseng

**Figure 2:**
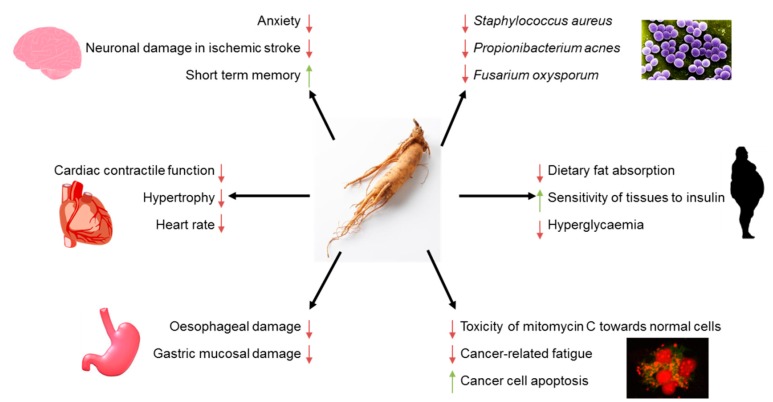
The main health beneficial activities of American Ginseng. It mitigates symptoms typical for Alzheimer disease, prevents neuronal damage in the course of ischaemic stroke and enhances cognitive performance—predominantly short-term memory. It depresses cardiac contractile function, decreases heart rate and diminishes hypertrophy. Furthermore, it attenuates oesophageal damage resulted from reflux oesophagitis and prevents the gastric mucosa from ulcer formation. It displays antimicrobial activity against different pathogenic strains. Its anti-obesity effect is mediated by lowering of dietary fat absorption. Moreover, it has anti-diabetes potential manifested by improvement of tissues' sensitivity towards insulin. Last, but not least, it exerts anti-cancer effects; its administration leads to apoptosis of cancer cells, helps to eliminate toxic effects of chemotherapeutics to healthy cells, and decreases cancer-related fatigue.

Influence of ginsenosides on human health has been extensively investigated over decades. Currently, it is one of the most commonly applied medical herbs all over the world [52]. AG exerts profitable effects on the functions of nervous, cardiovascular, and immune system [59,60,61]. Furthermore, variety of research showed activity of ginsenosides as anti-cancer and antimicrobial agents [62,63]. AG is considered an Adaptogen, a substance that enhances human health by restoration of homeostasis [10]. The main health beneficial activities of AG are presented in Figure 2.

### 4.1. Nervous System

Shin et al. observed that AG improved the status of mice with a model of Alzheimer-like syndromes [64]. In Alzheimer’s disease, amyloid-beta (Aβ) peptides promote damage of presynaptic cholinergic system and decrease in choline acetyltransferase (ChAT) activity. After application of AG extract (30–300 mg/kg bw), an increase of acetylcholine (AChE) production resulted from choline-acetyltransferase up-regulation in the brain of mice with β-amyloid was observed. Consequently, learning and memory functions were improved [64].

According to a general population-based survey, almost 33.7% of the world population is afflicted by an anxiety disorder at some point during their lifetime [65]. Wei et al. observed an anxiolytic effect of AG ginsenosides (50 and 100 mg/kg bw) in mice and compared it with that induced by diazepam [66]. AG saponins in contrary to diazepam did not influence locomotion abilities [66]. Furthermore, AG was shown to enhance cognitive performance in volunteers predominantly by improving working memory processes (200 mg dose) [67]. Liu et al. observed a neuroprotective effect of AG in the tsA-201 embryonic kidney cells transfected with cDNA expressing α subunits of the Brain_2a_ Na^+^ channel in ischemia [68]. Abnormal Na(+) flux in ischemia results in neuronal damage and AG extract (1 and 3 mg/mL dosages) reversibly blocked the channel in a dose-dependent manner. Ginsenoside Rg1, an active compound of AG as well as its primary and end metabolites Rh1 and protopanaxatriol (5–10 mg/kg bw), respectively, significantly diminished memory impairment in mice and increased hippocampal excitability of anaesthetized rats [69].

AG (10 mg/kg bw administered 4 times in a 2 h interval and in a dosage of 4 and 8 mg/kg bw twice in a 4 h interval) mitigated anxiety caused by methamphetamine administration in rats, shortened time of immobility in forced swimming test, and significantly decreased the number of errors in the maze test [70]. Results of some studies on AG activity in the nervous system are presented in Table 2.

### 4.2. Cardiovascular and Gastrointestinal Systems

Jiang et al. observed structural changes and cardiac function attenuation with no changes in blood pressure in hypertensive rats after administration of ginsenosides Rb1 and Rg3 (20 mg/kg bw) [78]. In another study, Jiang et al. observed that a single dose (300 mg/kg bw) of AG extract depressed the cardiac contractile function in rats, resulting in a reduced heart rate maintained for 24 h [79].

Ischemia and hypoxia associated with stroke result in cytokines release, glutamate excitotoxicity (nerve cells damage and death due to excessive stimulation by glutamate), oxidative stress, nerve cells apoptosis, invalid metabolism and inflammation [80]. Pre-treatment with *P. quinquefolium* PPD saponins dosage of (25 and 50 mg/kg bw) significantly increased the activities of ROS-regulating enzymes in the brain tissue of rats. These results indicate that because of its antioxidant properties, *Panax* extract might act protectively against stroke [80]. Administration of AG extract to rats inhibited hypertrophy and heart failure induced by isoproterenol through attenuation of cardiac adrenergic responses [81]. AG (50 mg/kg bw for 7 days) displayed a preventive effect against cardiomyopathy in mice, which was underlined by inhibition of oxidative stress [82]. It also protected mouse hearts from reperfusion injury through upregulation of inducible nitric oxide synthase (iNOS) expression [82].

Aqueous extract of AG inhibited superoxide generation in the heart of mice challenged with lipopolysaccharide to induce endotoxemia, a model of sepsis [83]. A similar effect was observed in cultured cardiomyocytes. Moreover, the extract inhibited the expression of NOX2, a main cardiac isoform of NADPH oxidase, a major enzyme producing ROS. It was concluded that AG inhibited the myocardial NOX2-ERK1/2-TNF-α signalling pathway. Antioxidant action of AG in cardiomyocytes was associated with the activation of nuclear factor (erythroid-derived 2)-like 2 (Nrf2) [4].

AG extract (50, 100, and 200 mg/kg bw) protected against oesophageal damage resulted from reflux oesophagitis through reduction of inflammatory cells and oxidative load in the oesophagus [84]. Another study demonstrated that AG (500 and 1250 mg/kg bw) protected rats from gastric mucosa damage induced by a chronic ethanol intake [85].

### 4.3. Anti-Cancer Activity

Cancer is one of the most common terminal diseases and is considered the second leading cause of death globally. According to World Health Organization it was estimated to account for 9.6 million deaths in 2018 [86]. Application of phytochemicals in cancer prevention and therapy is an emerging issue due to their availability and lack of adverse side effects [87,88,89,90,91]. AG has also been investigated in terms of its anti-cancer activity. Many studies on anti-cancer activity of AG focus on colorectal cancer. It represents approximately 19% of cancer incidence worldwide and accounted for 860,000 deaths in 2018 [86]. One of the most abundant AG constituents, ginsenoside Rh2, exerted a strong anti-tumor effect on colorectal cancer HCT116 cell line [92]. Ginsenoside Rh2 decreased proliferation of HCT116 cells through inhibition of PDZ-binding kinase/T-LAK cell-originated protein kinase, which is a serine/threonine protein kinase highly expressed in these cells [92]. HCT116 and HT-29 colorectal cancer cells were arrested in G1 phase of the cell cycle after incubation with the compound K, a gut microbiota metabolite of ginsenoside Rb1 [93]. Similar results were obtained by Gao et al. who showed that PPD inhibited HCT116 cancer cells proliferation through G1/S checkpoint arrest [94]. PPD inhibited tumor growth in xenograft HCT116 mice through the inhibition of the nuclear factor kappa-light-chain-enhancer of activated B cells (NF-kB) and c-Jun N-terminal kinase (JNK) signaling pathways [94]. AG inhibited formation of colitis in mice induced by azoxymethane/dextran sodium sulphate during and after exposure to the carcinogens along with reduction of activation of pro-inflammatory cytokines [95].

Water extract of AG (20, 40, 60, and 80 mg/mL) changed morphological and mechanical properties of human hepatocellular carcinoma SMMC-7721 cells that displayed an increased susceptibility to apoptosis [96]. AG was also found to display anti-cancer activity in human breast cancer MCF-7 cells through inhibition of their proliferation underlined by alterations in the mitogen-activated protein kinase (MAPK) signalling pathway [97].

A 4 hr-steamed AG root extract (S4h) (0.1 mg/mL) was reported to damage mitochondria, increase ROS level, and induce apoptosis in colorectal cancer cells [98]. S4h activated the NF-κB pathway, which was abandoned by the removal of ROS by antioxidants. Inhibition of the NF-κB pathway by a specific inhibitor increased the extent of S4h-induced apoptosis and a similar effect was observed when antioxidants were added. Both ROS levels and apoptosis were decreased when mitochondria were protected by ROS/NF-κB-mediated survival pathway. The final conclusion from that study was that ginseng induces apoptosis in colorectal cancer cells through mitochondrial damage, which may also activate the ROS/NF-κB-mediated survival pathway. 

Mitomycin C (MMC) is an antibiotic commonly applied as anti-cancer drug. Its activation leads to ROS generation, DNA alkylation, and formation of DNA interstrand crosslinks, as well as some chromosomal abbreviations [99]. Serious side effects of MMC greatly limit its use. Pawar et al. observed that AG root extract (50 and 100 mg/kg bw administered for 3 and 7 days) decreased the number of micronucleated polychromatic erythrocytes in mice pre-treated with MMC [100].

*Cis*-diamminedichloroplatinum (cisplatin) is a commonly used anti-cancer drug with nephrotoxicity as its main side effect [101]. Ma et al. showed that AG berry extract (AGBE) (300 mg/kg bw) decreased histopathological changes as well as increased levels of urea nitrogen and creatinine in serum of mice receiving cisplatin [102]. Pre-treatment with AGBE ameliorated oxidative stress induced by cisplatin. This was evidenced by a decrease in kidney malondialdehyde concentration, cytochrome P450 E1 (CYP2E1), renal 4-hydroxynonenal (4-HNE), and an increase in reduced glutathione and superoxide dismutase (SOD) content. Moreover, AGBE inhibited expression of TNF-α, interleukin-1β (IL-1β), cyxlooxygenase-2 (COX-2) and iNOS, proteins involved in the inflammatory response. Furthermore, pre-treatment with AGBE inhibited NF-κB and MAPK activated by cisplatin. Also, AGBE decreased the level of the apoptotic protein Bax, cleaved caspase 3 and cytochrome c released from mitochondria, but increased the level of anti-apoptotic Bcl-2. Therefore, renal oxidative stress, inflammation and apoptosis could be involved in cisplatin-related nephrotoxicity. The authors concluded that the protective effect of AGBE against nephrotoxicity induced by cisplatin was underlined by the ROS-mediated the NF-κB and MAPK signaling pathways. The potential of AGBE to ameliorate side effects of cisplatin was shown earlier by Mehendale et al. who also pointed at antioxidant ginsenoside Re as an AGBE component, which could be primarily responsible for the observed effects [103].

Ionizing radiation (IR) damages cells by a direct or indirect effect. The former results from absorption of a quantum of IR in the target site, leading to its ionization, whereas the latter is associated with water hydrolysis (radiolysis) and production of ROS, which damages biomolecules including DNA [104]. AG extract protected human lymphocytes irradiated with 1 or 2 Gy gamma irradiation in a dose-dependent manner [105,106]. This protective effect was manifested by a reduction in the number of micronuclei, a decrease in ROS levels, and an increase in total antioxidant capacity. The effects induced by ginseng were similar to those evoked by WR-1065, a biologically active amino thiol form of amifostine (WR-2721), the gold standard of chemical radio protectors. It is worth noting that this protective effect was observed at 90 min after irradiation. Therefore, ginseng can be considered not only as a preventive substance in radiological protection, but also as therapeutics in post-radiation disorders. It seems that the activation of NF-κB may be critical for this effect as it can interact with the JNK pathway to upregulate the pro-apoptotic Fas ligand. In their earlier work, these authors showed a key role of the mitochondrial pathway in AG cytotoxicity in colorectal cancer cells as many genes of that pathway were involved in apoptosis of SW-480 colon cancer cells [107].

One of the most unwanted symptoms related to cancer and cancer therapy is cancer-related fatigue (CRF). It is defined as a relentless, subjective feeling of tiredness not related to activity that affects normal functioning [108]. It was documented to occur among patients during treatment and in patients that already completed the therapy [109]. It was shown that application of AG resulted in a decrease of CRF and a more pronounced effect was observed in patients during active cancer treatment than those who had completed therapy [110]. Some preclinical data may partly explain observed effects as AG downregulated inflammatory pathways, decreased inflammation and modulated cortisol levels in experimental animals and cell cultures, and these effects can be related to CRF [97,111,112,113].

### 4.4. Anti-Diabetic Activity

**Figure 3:**
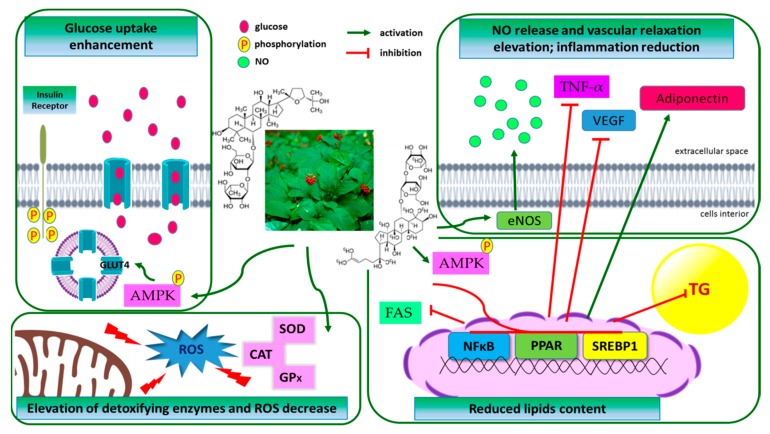
Proposed molecular mechanisms of anti-diabetic and anti-obesity actions of American Ginseng. See main text for more details. AMPK-AMP-activated protein kinase; CAT—catalase; eNOS—endothelial nitric oxide synthase; FAS—fatty acid synthase; GLUT4—glucose transporter 4; GPx—glutathione peroxidase; NF-κB—nuclear factor κB; NO—nitric oxide; PPAR—peroxisome proliferator-activated receptor; ROS—reactive oxygen species; SOD—superoxide dismutase; SREBP-1c—sterol regulatory element binding protein 1c; TG—triglyceride; TNFα—tumour necrosis factor α and VEGF—vascular endothelial growth factor.

Abnormalities in the regulation of glucose metabolism in type 2 diabetes mellitus (T2DM) subjects lead to postprandial chronic hyperglycemia. After meal intake, glucose is released to the blood due to the activity of carbohydrate or oligosaccharide digestive enzymes, such as α-amylase and α-glucosidase. Systemic studies performed by Kan et al. showed that in cell-free assays AG root extract had no inhibitory activity on α-glucosidase and α-amylase [114]. In the same study these authors showed in vitro that AG increased glucose uptake and insulin sensitivity in differentiated adipocytes, which in turn may affect the hypoglycemic effect (Figure 3). Further research carried out on Sprague Dawley rats presented that mixture of AG root, *Morus alba* leaf and *Trigonella foenum graecum* seed preparations elevated glucose uptake and insulin sensitivity in mice 3T3-L1 adipocytes via an increase of the expression of glucose transporter 4 (GLUT4). GLUT4 transports glucose across the plasma membrane and its activity is regulated by insulin. Furthermore, accumulating data indicate that oxidative stress resulted from excessive ROS production or/and reduced antioxidant defense is involved in the pathogenesis of chronic blood glucose increase and insulin resistance. It was observed that chronic ginseng consumption by old (22 months) rats decreased the levels of ROS in heart, soleus muscle, and deep portion of the *vastus lateralis* muscle [115]. These animals also showed a higher activity of glutathione peroxidase (GPx) and decreased level of carbonyl group. It was concluded that ginseng consumption might ameliorate oxidant production and oxidative damage to proteins mainly by the increase in the activity of antioxidant enzymes. Ginseng in general is known to reduce blood glucose levels [116]. Yoo et al. showed that in diabetic db/db mice treated for 30 days with a steamed AG roots preparation at 150 mg/kg bw the hepatic glycogen and plasma insulin levels were significantly elevated, while blood glucose was lowered [117]. Comparing to untreated, steamed AG preparation possessed higher antioxidant activities due to the increased level of ferulic and cinnamic acids and elevate liver SOD and GPx. That observation is in line with the earlier report showing that AGBE and ginsenoside Re reduced oxidative damage in MIN-6 pancreatic β-cell line induced by acute or chronic incubation with hydrogen peroxide [118]. It was concluded that the anti-diabetic effect of AGBE could be attributed to its antioxidant properties and the Re ginsenoside might significantly contribute to that effect. Furthermore, steamed ginseng preparation considerably increased high density lipoprotein (HDL) concentrations, while levels of plasma cholesterol and low density lipoprotein (LDL) were significantly decreased [119].

Most studies on the beneficial properties of ginseng are performed on extract from its root. Also, ginseng herbal supplements usually contain extract from the root. However, it was reported that different ginseng parts contain different chemical compounds with a potentially different biological activity [119]. AGBE presents different chemical content than AG root extract and this difference also concerns distinct ginsenoside compositions [120]. Shao et al. showed that AGBE directly scavenged hydrogen peroxide and superoxide anions [121]. They also showed that AGBE (0.1, 0.5 and 1 mg/mL) displayed a protective effect against acute oxidative stress induced by hydrogen peroxide and antimycin A (an inhibitor of mitochondrial complex III of the mitochondrial electron transport chain) in cultured chick cardiomyocytes. This was extended by showing that Re, the main ginsenoside of AGBE, produced essentially the same effect [122]. Therefore, both extracellular and intracellular oxidative stresses were tested and both were inhibited by AGBE. These results were supported by a subsequent work of these authors, which showed the protective effects of AGBE against chronic oxidative stress induced by hydrogen peroxide [123]. The authors concluded that this effect was associated with upregulation of peroxide detoxifying mechanisms, including catalase activity. 

Accumulating data confirm that diabetic blood glucose fluctuations strongly influence the development of cardiovascular complications via induction of endothelial cells dysfunction and apoptosis [124]. It was already shown that AG*,* apart from the direct scavenging of radicals, could be involved in endothelium-dependent release of NO and its inactivation [123]. It is known that increased quantities of ROS react directly with NO and produce cytotoxic peroxynitrite. Studies performed by Wang and al. demonstrated a lowered production of serum NO, ET-1 (endothelin-1), TNF-α and sICAM-1 (soluble intercellular adhesion molecule 1) in rats with streptozotocin-induced diabetes on high fat and high caloric diet treatment with ginseng stem and leaf saponins for 10 weeks (30–60 mg/kg bw) [124]. The authors showed that AG had comparable efficiency to metformin, a known anti-diabetic medicine, in vessel stress relief and inflammatory reaction reduction. Sen et al. observed a beneficial effect of alcoholic extract of AG on diabetic complications in mouse models of type 1 (C57BL/6 mice with streptozotocin-induced diabetes) and type 2 diabetes mellitus (db/db) [125]. The Rb1 and Re ginsenosides dominated in this extract. Authors observed that ginseng administration improved dysmetabolic state in diabetes animals. Ginseng prevented oxidative stress assessed by the accumulation of superoxide anion and mRNA expression of the heme oxygenase 1 (HO-1) gene, and upregulated proteins involved in vessels architecture in the heart and the retina of diabetic mice. Cardiac functions were improved in animals receiving ginseng. The authors attributed this beneficial influence of ginseng on diabetic complications to its antioxidant, and to a lesser degree to its antihyperglycemic action. 

Advanced studies performed on T2DM patients with concomitant hypertension showed that AG root preparation (3 g/day) after 3-month supplementation improved arterial stiffness and systolic blood pressure [126]. Aside from that observation, it was shown that the root preparation had no influence on blood pressure in healthy subjects. According to Xu et al., observed vasodilatory effect may be a consequence of increased NO generation after AG treatment [127]. Glucose-induced oxidative stress and damage in human umbilical vein endothelial cells (HUVECs) were prevented by incubation with ginseng root, resulting in lowering of NF-κB, fibronectin and vascular endothelial growth factor (VEGF) mRNA and protein levels [128]. In hyperglycaemic conditions, overproduction of fibronectin decreases motility of cells and their replication; elevation of VEGF leads to endothelial dysfunction due to the enhancement of capillary formation and cell permeabilization, while the NF-κB activation in turn exaggerates the expression of extracellular proteins, like fibronectin and vasoactive factors (i.e., VEGF). Moreover, AG preparation acted preventive against glucose-induced oxidative DNA. 

In vivo research performed by Amin et al. on rats with streptozotocin-induced diabetes showed that animal treatment with AG (at 300 mg/kg bw for 10 days) resulted in increased levels of β-cells insulin and C-peptide secreted by pancreas [129]. Their studies presented more detailed mechanism of AG anti-diabetic activity with the involvement of hepatic gluconeogenic glucose-6-phosphatase (G6Pase) and glycogenolytic liver glycogen phosphorylase inhibition.

Because AG has been used as an alternative medicine for a long time to support its clinical efficacy in glycemic control a few randomized controlled trials were performed. Vuksan et al. were the first that assessed short-term effect of AG root on postprandial glycaemia in human nondiabetic subjects and subjects with T2DM [130,131]. It was revealed that 3 g of root ginseng preparation administered together with the glucose challenge (25 g) attenuated postprandial glycaemia by 20% in both, healthy and diabetic subjects, in a comparable way. However, preparation given 40 min prior to glucose challenge lowered glucose concentration only in diabetic patients. Further studies performed on healthy subjects treated with 3 g of root alcohol extract revealed no influence of preparation on postprandial glycaemia and insulin level [132]. On the other hand, the authors observed that 50% extract possessed insulin-sensitizing effects in nondiabetic humans, improving their metabolic parameters. What is more important, advanced clinical studies performed for 12 weeks on T2DM patients with root extract at a dose of 3 g/day pointed its safety against liver, kidneys and hemostatic functions damage [133]. Recently, the clinical trial coordinated by Vuksan et al. demonstrated that patients dosed with 3 g of root preparation/day after 4-week treatment displayed significantly reduced levels of glycated hemoglobin HbA1c (by ≥ 0.3%), fasting blood glucose (0.71 mmol/L) and systolic blood pressure (by 5.6 ± 2.7 mmHg), although the insulin release was increased by 1.5 fold. The elevation of NOx production (1.85 ± 2.13%) reflected ginsenosides stimulated improvement of endothelial function [134]. These outcomes are followed by present research, where different combinations of AG and other potent hypoglycemic preparation—konjac-based fiber blend (KGB)—were used as ingredients of formulas for future medicines [135]. The synergic effect of mixture (6 g of fibre from KGB: 3 g of AG per day) was obtained with clinically significant reduction in glycated hemoglobin HbA1c (0.31%) and lipids concentrations (8.3 ± 3.1% in cholesterol carried within low-density lipoprotein particles (LDL-C), 7.5 ± 2.4% in non-HDL cholesterol (non-HDL-C), 5.7 ± 1.9% in total cholesterol (total-C), 4.1 ± 2.1% in total-C:HDL-C ratio) over 12-week treatment of T2DM patients.

### 4.5. Prevention of Obesity

Low physical activity and elevated energy intake lead to excessive fat accumulation in the body (especially in adipose tissue) and weight gain. Obesity increases the risk of T2DM, cardiovascular complications, and certain types of cancer [136]. Whereas hypoglycemic activity of AG is quite well- known, few studies address its anti-obesity properties. Therefore, the effect of AG on the regulation of lipid metabolism is still not fully understood (Figure 3).

Intestine absorption of dietary fat, and finally reduction of weight and hyperlipidemia, could be lowered by inhibition of pancreatic lipase activity during digestion. That enzyme is responsible for the hydrolysis of majority of dietary fats. It was shown that crude saponins isolated from AG stem and leaves acted as lipase inhibitors lowering the rate of oleic acid release from triolein [137]. The Rc, Rb1 and Rb2 ginsenosides were identified to be mainly responsible for observed effects. The same saponins extract at dosage of 1 g/kg bw prevented the plasma triacylglycerol elevations in rats after oral intake of lipid emulsion and prevented high-fat diet-induced fat storage in adipose tissue. More recently, Liu and co-workers found that PPD types of saponins isolated from AG leaves inhibited the porcine pancreatic lipase activity in vitro in a dose-dependent manner (0.25–1 mg/mL), whereas PPT showed no inhibitory activity [138]. Furthermore, mice fed with high-fat diets containing 0.02 or 0.05% PPD had lower adipose tissue weights, limited accumulation of hepatic triacylglycerol and total cholesterol levels. 

Increased number of preadipocytes (hyperplasia) and the increased size of adipocytes (hypertrophy) are the main metabolic by-products of adipose tissue modelling. As cytokines and adipokines are released from adipose tissue, adipogenesis (preadipocyte differentiation) plays a key role in obesity development [139]. Studies performed with mice preadipocyte 3T3L1 cells showed that AG ethanol extract containing Rg1, Re, Rb1, Rc, Rb2, and Rd ginsenosides exerted a dose-dependent effect on cells proliferation (LC_50_ 40.3 ± 5 μg/mL) with reduction of preadipocyte cell growth and intracellular lipid accumulation. Research also revealed an upregulation of adiponectin, the key regulator of insulin sensitivity [140]. Experiments carried out with Rh2 and Rg3 ginsenosides identified in AG preparation revealed that these compounds effectively inhibited adipogenesis. That process was accompanied by the activation of AMPK, which is involved in lipid and cellular energy regulation, as well as inhibition of peroxisome proliferator-activated receptor-γ (PPAR-γ), a nuclear receptor. These proteins are recognized as the main regulators of adipogenesis and lipogenesis through their ability to change the expressions of CCAAT/enhancer binding protein α (C/EBP-α), adiponectin and leptin, fatty acid binding protein 4 (FABP4), GLUT4, acetyl-CoA carboxylase (ACC), fatty acid synthase (FAS) and perilipin [136]. After adipogenesis induction the expressions of sterol-regulatory-element-binding protein 1 (SREBP1), that is a downstream adipogenic transcriptional regulator of lipogenic genes, was upregulated. However, there is evidence of different mechanism of AG ginsenosides influence on adipogenesis: pseudoginsenoside F11, that is found in AG and not in *P. ginseng*, promoted adiponectin secretion, fat accumulation and activation of PPAR-γ in 3T3-L1 cells [141]. Interestingly, the authors found that simultaneously pseudoginsenoside F11 inhibited obesity-linked phosphorylation of PPAR-γ at Ser-273 by Cdk5, which in turn disrupted the contact between PPAR-γ and retinoid X receptor RXR𝛼, which eventually disrupted PPAR-γ/RXRα heterodimer formation and its binding to DNA. Furthermore, Wilson et al. showed that extraction technique determined biological activity of AG preparation [142]. It was observed that water AG extract containing polysaccharides acted as a strong inflammatory modulator, increasing the expression of *Il-6*, *NF-κB* and *TNFα* in adipocytes, whereas the ethanol extract had no such influence. Further studies demonstrated that AG water extract caused a significant increase in the expression of inflammatory genes (*Mcp1, TNFα, Nos2, Ccl5, Il6*) not only in adipocytes, but also in other cells type co-cultured in the presence of adipocytes, like murine macrophages RAW264.7 [143]. Recently, Singh et al. showed beneficial properties of AG ethanol extract on mice deficient in the *Pcyt2* (ethanolamine-phosphate cytidylyltransferase 2) gene (ETKO/Pcyt2+/-) [144]. ETKO/Pcyt2+/- mice are able to develop fatty liver, obesity, and hypertriglyceridemia, as well as insulin resistance at the age of 25–30 weeks, and these dysfunctional changes are similar to those occurring in humans. Mice treated with AG (200 mg/kg bw for 24 weeks) reduced the fatty liver, hepatic, and intestinal lipoprotein secretion and circulating lipids. More detailed analysis revealed that this was accompanied by a reduction of the expression of the *SREBP1*, *PPARα* and *FAS* genes in liver. These changes were accompanied by an elevation of AMPK protein level, but without affecting phosphorylation of ACC involved in malonyl-CoA for fatty acid synthesis. AG preparation had no influence on the intestinal expression of fatty acid translocase *CD36* and fatty acid transport protein 4 (FATP4) genes in control subjects; however, it downregulated these genes in ETKO/Pcyt2+/- mice intestine. 

### 4.6. Anti-Aging Properties

No controlled clinical trials on the influence of AG on life expectancy were performed. Many studies on its effects on both chronological and biological aspects of ageing relate them to antioxidant properties of AG. 

AG (2.25 g/kg for 14 days) was hypothesised to protect against premature ovarian failure (POF), a loss of ovarian function before 40 that can be considered as a marker of accelerated ageing [145]. That study showed that AG protected against POF by regulating prostaglandin biosynthesis, ovulation, and preventing ovarian ageing.

Fernández-Moriano et al. showed that Rb1, the main ginsenoside of AG, demonstrated neuroprotective potential in SH-SY5Y cells that are a model for neurodegenerative disorders [146]. This effect was manifested mainly by antioxidant action of the ginsenoside: ROS scavenging, increasing the activity of the glutathione system and SOD and activation of the Nrf2 pathway. Moreover, the protective effect against mitochondrial dysfunction was observed, which further supports antioxidant properties of Rb1 and altogether suggests its potential influence on the pathogenesis of neurodegenerative diseases and in general mitochondria-dependent ageing processes. 

Murphy et al. showed that powdered root of AG (100 mg/kg bw for 14 and 28 days) stimulated copulatory behavior of male rats expressed by a decrease in mount, intromission and ejaculation latencies [147]. There were no differences between ginseng-treated and control animals in plasma luteinizing hormone and testosterone, but plasma prolactin levels decreased in the treated rats. It was suggested that ginseng might induce changes in dopaminergic neurotransmission that could contribute to the observed stimulatory effects.

The incidence and prevalence of stroke in general and ischemic stroke in particular increase with age and some features of ischemic stroke are age-related [148]. Brain damage resulting from ischemic stroke is mediated by disturbed flux of Na^+^ ions and drugs blocking voltage-dependent sodium channels are used to protect neurons and other cells during ischemic episodes [149]. Liu et al. showed that water extract of AG and its main ginsenoside Rb1 tonically and reversibly blocked the brain in transformed human kidney tsA201 cells transfected with Brain_2a_α subunits of the voltage-dependent sodium channel [68]. As effects induced by both whole root extract and isolated Rb1 were comparable, the authors concluded that Rb1 might be primarily responsible for the observed effects. Ginseng and Rb1 induced qualitatively similar effects as lidocaine, a known Na+ channel blocker, so it was concluded that the observed blockade was due to the interaction of ginseng/Rb1 with the inactive state of the channel.

Multiple sclerosis (MS) belongs to the most serious of problems in contemporary medical care. Its mechanism is not fully known, but its pathogenesis is associated with overproduction of ROS released by microglia and immune cells infiltrating the central nervous system [150]. These ROS may demyelinate and otherwise damage axons. It was shown that water extract of ginseng (150 mg/kg bw for 14 days) reduced the clinical signs of experimental autoimmune encephalomyelitis (EAE) in mice, an animal model of MS [72]. Ginseng also reduced the extent of demyelination. This improvement of MS-related symptoms was associated with effects, which could contribute to anti-oxidative action of ginseng: decrease in the activity of immunoreactive iNOS and levels of circulating TNF-α. As MS is ageing-related disease it can be speculated that the effects of ginseng may be underlined by its general anti-aging action.

Alzheimer’s disease (AD), similarly to MS, is a chronic neurodegenerative disease affecting primarily the elderly with no effective treatment available [151]. In AD, the presynaptic cholinergic system is degenerated with the involvement of amyloid-beta (Aβ) peptides, resulting in decreased activity of ChAT and, consequently, diminished levels of AChE. It was observed that an extract from AG containing high concentration of Rb1 ginsenoside protected human neural stem cells from toxicity induced by synthetic Aβ_1-42_ peptide [64]. The extract also restored the expression of the *ChAT* gene suppressed by Aβ_1-42_. Injection of the peptide into the brain of experimental mice worsened their cognitive function that were recovered by oral administration of the ginseng extract, which also restored some biochemical markers disturbed by Aβ_1-42_ injection: microtubule-associated protein 2 (MAP2), synaptophysin and AChE. Therefore, the beneficial action of ginseng might be associated with restoring neuronal integrity disrupted by Aβ peptides. Decreased levels of synaptophysin, observed in AD patients, are associated with impaired cognitive functions [152]. It is not completely clear whether the protective effect of ginseng against Aβ_1-42_ peptide was attributed to its degradation or ameliorating of effects already induced by the peptide. The mechanism of the former effect can be partly explained by the observed increase in the activity of neprilysin, an Aβ-degrading enzyme, by Rg3 [153]. However, Al-Hazmi et al. reported that ginseng extract (100 and 200 mg/kg bw for 14 days) improved memory through recovery of AChE and restoration of other functions in rat brain [154]. Therefore, further study is needed to determine the potential effects of ginseng in AD through direct degradation of Ab peptides and ameliorating of effects of their detrimental action.

AG extract (administered in dose 8 g/kg for 12–33 days) was shown to improve some cognitive functions affected by ageing in rats [155]. Free-radical theory of ageing says that ageing is associated with increased ROS production and these ROS damage different tissues leading to the accumulation of abnormalities and progressive deterioration of basic vital functions [156]. However, contemporary theories of ageing do not consider ROS overproduction as the decisive aspect of ageing, but ROS still play an important role in ageing as both the signalling molecules and a damaging/interfering factor [157]. 

Scholey et al. showed AG extract (doses 100, 200 and 400 mg) to enhance neurocognitive functions which was manifested by an improvement in working memory performance in an acute, randomised, placebo-controlled, cross-over study. No effect on blood glucose level was observed [68]. It was suggested that profitable effects of ginseng root on memory are due to its main ginsenoside Rb1 that could induce such effects by improving cholinergic metabolism. These studies were supported by Shi et al. who demonstrated a beneficial effect of AG on the neurocognitive functions in the SAMP10 substrain of senescence-accelerated mice (SAM) [158]. These mice show features of accelerated ageing, including cognitive dysfunctions and loss of neocortical synapses, which can be involved in intellectual decline in aged people. Enhancement in neurocognitive functions in SAM mice was attributed to the upregulation of insulin and choline acetyltransferase.

All of this data serve as rationale for further study of the potential modulation of both chronological and biological aging by AG.

## 5. Interactions of American Ginseng with Microorganisms

### 5.1. Antimicrobial Action

Sienkiewicz et al. compared efficiency of different parts of the plant: leaves, stalks, hairy roots and field roots. Results of the trial showed that ginsenosides originated from AG (minimal inhibitory concentration—MIC values from 0.5 to 1.7 mg/mL) possessed anti-staphylococcal activity. Moreover, antibiotic resistance in Staphylococcus aureus did not influence this feature. It was noted that not only leaves and field roots, but also hairy root cultures are a source of active compounds of AG [159]. Study conducted by Wang et al. [160] indicated antimicrobial activity of AG against two strains of *Propionibacterium acnes* (MIC 64 and 128 μg/mL) and *Staphylococcus epidermidis* (MIC 4.1 mg/mL). Less polar ginsenosides, including Rg2, Rg3, Rg6/F4, Rs3, and Rg5/Rk1 exerted greater antimicrobial activity than their polar counterparts (Rg1, Re, Rb1, Rc, Rb2, Rd). These results suggest that less polar fraction of ginsenosides might be used to produce innovative type of antimicrobial agents, including skin care products for prevention and treatment of acne [160]. Kochan et al. showed antimicrobial potential of AG against various pathogenic bacterial and yeast strains [64]. Extracts obtained from three different clones of AG hairy roots inhibited growth of *S. aureus*, *Enterococcus* spp., *E. coli*, *Pseudomonas aeruginosa* (MIC values from 0.8 to 1.4 mg/mL) and yeasts belonging to *Candida albicans* species (MIC values from 1.0 to 1.4 mg/mL). The strongest effects were observed for Gram negative *E. coli* strains. *Enterococcus* spp. was the most resistant to activity of extracts [64]. Alipour et al. showed similar effect in the *P. aeruginosa* O1 strain. AG extract (1.25–5% *w*/*v*) detached bacteria biofilm in microplates and led to reduction in number of living cells [161].

Xue et al. showed an inhibitory effect of AG ginsenosides on *Fusobacterium nucleatum*, *Porphyromonas gingivalis*, *Porphyromonas endodontalis* and *Prevotella intermedia* that may be involved in halitosis [162]. The effect was more pronounced for less polar ginsenosides that could destroy bacterial cell membrane more easily than they more polar counterparts. Apart from that a homodimeric protein quinqueginsin isolated from the root of AG exerted various antifungal activities against *Fusarium oxysporum*, *Rhizoctonia solani* and *Coprinus comatus* as well as degraded tRNA in yeasts. It also demonstrated antiviral activity by inhibition of human immunodeficiency virus-1 reverse transcriptase [163].

### 5.2. Metabolism of American Ginseng Ginsenosides by Intestinal Microbiota

Human intestinal microbiota has a significant impact on the metabolism of ginseng saponins. After ingestion, ginsenosides undergo extensive biotransformation [164]. Hydrophilic constituents are metabolized to hydrophobic compounds. The absorption of the metabolites increases with the activity of faecal gut microbiota responsible for metabolism of ginseng saponins. Those metabolites, e.g., compound K, can display higher activity than parental substances [165]. Wan et al. showed the presence of 25 metabolites of ginsenosides from AG root extract mixed with intestinal microbiota isolated from human faeces [166]. Fifteen of them were derivatives of original PPD saponins, 7—metabolites of PPT and remaining 3 of oleanolic acid. These results indicate that the PPD-type ginsenosides were metabolized more efficiently than others. Main metabolic pathways were deglycosylation performed by sequential cleavage of sugar moieties and dehydration. The most frequently found metabolites were ginsenoside Rg3, F2 and compound K. Another study determined metabolites of ginsenosides in human plasma, urine and faeces after ingestion of AG extract by male volunteers [167]. By means of liquid chromatography coupled with quadrupole time-of-flight mass spectrometry 15 peaks were observed in plasma. Ten peaks represented parental ginsenosides and 5—their metabolites. Ginsenosides 20*S*-Rg2, 20*R*-Rg2, F2, 20*R*-Rg3, and compound K were the most abundant in plasma. Twenty peaks were observed in urine; 10 of original ginsenosides and 10 of metabolites. Ginsenosides 20*S*-Rg2, 20*R*-Rg2, 20*S*-Rh1, 20*R*-Rh1, F1, F2, 20*R*-Rg3, 20*S*-Rh2, 20*R*-Rh and compound K were the major metabolites found in urine. In faeces as many as 36 peaks were detected. Twenty of them represented original ginsenosides and 16—their metabolites. Therefore, metabolism of ginsenosides may play a crucial role in their bioactivity. Compound K, the main metabolite of the PPD group, displays higher activity than its parental compound Rb1 [167].

An AG fortified yogurt containing probiotic *Lactobacillus rhamnosus* GR-1was developed to improve the beneficial effects of AG [168]. It was observed that in the case of bacteria culture with applied aqueous ginseng extract viability of *Lb. rhamnosus* GR-1 was greater in comparison to the culture without AG extract during the 28-day storage period. The results suggested that aqueous AG extract maintained a synbiotic relationship with probiotic strain of *Lb. rhamnosus* GR-1 [168].

AG influence on gut microorganisms was investigated in mice with chemically induced colitis [169]. This study showed that AG decreased the growth of bacteria belonging to Bacteroidetes and Verrucomicrobia and increased growth of Firmicutes, suggesting that AG could restore composition of normal gut microbiota disturbed by pathological processes associated with colitis formation.

## 6. Conclusions and Perspectives

AG has been applied for centuries as a diet supplement due to a chemical composition rich in active compounds: ginsenosides. These saponins belonging to a group of glycosides are responsible for wide range of health-beneficial activities of the plant and may allow to distinguish AG from other *Panax* species. AG is also rich in unsaturated fatty acids, linoleic and linolenic, which are important for proper function of the human organism. Different parts of the plant, the leaves, stem, flowers, berries and primarily roots (raw or thermally processed), can be a raw material for production of extracts. There are many commercial products containing AG extracts, including shredded slices, drinks, alcoholic beverages and dried sugar coated roots. Also, AG is used in cosmetics such as shower gels, hair conditioners, lotions, and shampoos [28].

This review highlights the most important characteristics and possible applications of AG. Among numerous studies AG was documented to exert beneficial activity towards nervous system. It boosts memory, increases calmness, and enhances cognitive performance [68]. It has therapeutic potential in treatment of Alzheimer disease and anxiety [64,66]. It also affects cardiovascular system—changes cardiac structure in hypertension, reduces heart rate, inhibits hypertrophy and heart failure [79,81,128,170]. Furthermore, AG prevents oesophageal damage resulted from reflux oesophagitis and formation of ulcer in gastric mucosa [84,85]. Apart from that, it displays antimicrobial activity against different pathogenic strains of bacteria including resistant to antibiotics *S. aureus* strain [159]. AG is extensively investigated in terms of anti-cancer activity. It promotes apoptosis of cancer cells and alters many different signalling pathways important for cancer transformation [92,93,94,95,96,97,98,171]. It also protects normal cells from unwanted side effects of anti-cancer drugs and diminishes cancer-related fatigue [100,110]. However, the mechanisms underlying beneficial activity of AG in cancer is not fully understood. Administration of AG may be beneficial for individuals suffering from obesity and diabetes. It enhances sensitivity of tissues to insulin and inhibits formation of adipose tissue. Also, polysaccharides of AG were reported to display pro-health effects by stimulating the immune system. Assinewe et al. observed that polysaccharide of AG extract containing glucose, galactose, arabinose, rhamnose, and mannose displayed cytokine-stimulating activity on macrophages. In this study, aqueous extracts of AG roots (1–100 μg/mL) stimulated the release of immunoreactive TNF, contributing to the activation of macrophages [172]. Yu et al. also showed immunostimulatory effect of the fraction of polysaccharide present in AG extract (50–400 μg/mL). AG extract application led to enhancement of splenic lymphocyte proliferation, macrophage phagocytosis and nitric oxide production [173]. AG extract (125 mg/kg for 3–6 days) stimulated alveolar macrophages in rats associated with increased level of NO, TNF-α and IL-6 in plasma [174]. These results were in agreement with those obtained by Azike. Similarly, AdG water extract (125 mg/kg) administered to rats for 3–6 days upregulated nitric oxide, TNF-α and IL-6. Within in vitro study macrophages were incubated with different concentrations of AG. A dose-dependent immunostimulatory activity of aqueous and alcohol extracts of AG (50–200 μg/mL) in rat macrophages was observed and an in vitro effect was more pronounced than corresponding in vivo result due to relatively low bioavailability of the extract administrated orally [175]. As the methodology of AG separation is poorly developed, it is a promising direction for the future research.

Although many health beneficial pharmaceutical effects have been attributed to AG, molecular mechanisms underlying these effects are poorly known. Usually, studies showing a biological/medical effect of AG or its component, suggest more than only one molecular target. This follows from the fact that pharmaceutical effects of AG may be underlined by several mechanisms of its action with many possible molecular targets linked with each of the mode of action. Therefore, we have not attempted to comprehensively address the problem of identifying molecular targets of AG as the length of this review is limited. Excellent reviews on this subject can be found elsewhere [9,10,11,12,176,177]. 

Most health effects of AG are mainly due to its strong antioxidant properties. In general, AG is frequently considered to have a greater capacity for free radicals scavenging and inhibit lipid peroxidation than its Asian counterpart [178]. To investigate mechanisms beyond antioxidant activity of AG, Kitts et al. showed that its extract containing main ginsenosides was able to scavenge DPPH (2,2-diphenyl-1-picrylhydrazyl)-stable free radical as well as and peroxyl (LOO•) and hydroxyl (•OH) free radicals [179]. Moreover, the extract efficiently chelated transition metals, preventing their involvement in the Fenton reaction, which was confirmed by inhibition of the induction of DNA strand breaks and suppression of protein oxidation. Additionally, the extract displayed protective action against lipid peroxidation in a metal-free solution. This important study shows the potential of AG to exert an antioxidant action by scavenging free radicals, chelating Fenton metals and preventing lipid peroxidation to further investigations in biological systems. These results were confirmed in a significant range in V79-4 Chinese hamster fibroblast-like cell line incubated with steamed root (red ginseng) of AG [26].

In spite of wide range of beneficial activities towards human organism, AG is not recommended for women during pregnancy and lactation period, because its teratogenic effects in rats and mice embryos as well as its not reported safety in breast-feeding women [180,181]. Because it has a cooling effect on the body, it is recommended to be consumed in summer [167]. Overdose of AG extract may lead to appearance of side effects such as insomnia, nervousness, gastrointestinal track disorders and symptoms of depression [27]. Components of AG extract may demonstrate antagonistic activity on drugs, such as anticoagulants, prescribed for chronic atrial fibrillation, mechanical valves, deep vein thrombosis and recurrent stroke [171]. It is difficult to determine the effective preventive and therapeutic concentrations of AG, because different units (e.g., mg/kg bw, mg/mL, mg) and time of application (e.g., 4 times in 2 h interval; twice in 4 h interval; for 12 h; for 2, 10, 12, 14, 30 or 33 days; for 10 or 24 weeks; for 3 months) are used in different studies. For this reason, it is not possible to make a comprehensive summary of effective preventive and therapeutic doses of AG, and it needs standardisation.

Currently, AG is a promising source of health beneficial phytochemicals. In view of its foregoing pro-health activity, possible mechanisms responsible for its mode of action and potential of application in different branches of medicine are worth further investigation. There are very few studies on AG interactions with probiotics in terms of its stimulatory or inhibitory activity on growth of certain strains of these beneficial microorganisms [165]. Apart from that, there is not much information about AG interactions with normal human microbiota, its influence on ginsenosides transformation, and their biological activity in the human body [165,166,167]. The antibiotic effect of AG towards pathogenic strains also requires more extensive investigation [159,160,161,162,163]. American Ginseng still has great potential to be explored in both basal research and clinical medicine.

## Figures and Tables

**Table 1:** Main ginsenosides of American Ginseng and their pharmacological activity.

Pharmacological Action	Ginsenoside	Reference
Positively affects memory processes; induces synthesis of acetylcholine in the hippocampus by stimulating choline acetyltransferase; induces apoptosis and inhibits angiogenesis in cancer cells; inhibits the release of inflammatory leukotrienes; reversibly and tonically blocks voltage-dependent Na+ channels in the brain reducing detrimental effects of hypoxia; downregulates the *COX-2* gene; stabilises neutrophils and lymphocytes; inhibits the release of histamine; blocks calcium channels and stabilised the heart; reduces blood sugar levels; anti-diabetic, insulin-sensitising and anti-obesity actions; neurotropic, neuroprotective, oestrogen-like activity; stimulates GABA receptors and induces a depressive effect on brain function, which underlines its calming, anxiolytic, sleeping, relaxing and antipsychotic effects	Rb1, Rb2, Rc	[9,22,53,54,55]
Stimulates superoxide dismutase; inhibits angiogenesis in cancer; prevents diabetes; lowers cholesterol and triglycerides levels, activates lipolysis; corticotropic and oestrogenic activity	Rb2	[9,54,55]
Inhibits proliferation of breast cancer cells; induces corticotropic effects	Rc	[9,22,54]
Promotes neurites outgrowth, an important process for neuronal repair; induces corticotropic effects	Rd, Rc, Re	[9,22,54,55,56,57]
Scavenges hydroxyl radicals and degrades H_2_O_2_; reduces blood sugar levels; induces cardioprotective effects; activates cGMP and relaxes smooth muscles	Re	[9,22,54,55,56]
Downregulates the *COX-2* gene; stabilises neutrophils and lymphocytes; inhibits histamine release; inhibits platelet-induced activation of thromboxane; increases insulin receptors; increases T-helper lymphocytes; inhibits release of endothelin and relaxation of the smooth muscle of blood vessels; activates cyclic guanosine monophosphate and relaxes the smooth muscle (hypotensive effect); blocks calcium channels and stabilised the heart; reduces blood sugar levels	Rg1	[22,55,56]
Inhibits neuronal acetylcholine	Rg2	[22]
Inhibits platelet aggregation induced by thrombin; relaxes the smooth muscle of the blood vessels by activating the K+ channels and releases Ca^2+^; inhibits progression of tumours and reduces drug resistance of cancer cells; inhibits endothelin and relaxation of the smooth muscle of blood vessels; induces hypotensive effect; downregulates the *COX-2* gene; stabilises neutrophils and lymphocytes; inhibits histamine release; modulates mitogen-activated protein kinases, thus inhibiting the spread of cancer cells	Rg3	[22,53,55,56,58]
Activates oestrogen receptor; inhibits proliferation of cancer cells and induces apoptosis	Rh1	[22,58]
Inhibits breast, liver and prostate cancer cells proliferation	Rh2	[22,58]
Assists memory improvement; induces neuroprotective effects	Pseudoginse-noside F11	[22]

**Table 2:** Effects of American Ginseng in the nervous system.

Effect	Reference
Mitigation of symptoms of Alzheimer disease in mice by AChE-acetyltransferase upregulation	[64]
Anxiolytic effect without influence on locomotion abilities in mice	[66]
Improvement of cognitive performance, predominantly working memory and calmness in young adults	[67]
Neuroprotective effect on tsA-201 cells transfected with cDNA expressing α subunits of the Brain_2a_ Na^+^ channel during ischaemia by blocking Na^+^ channel	[68]
Rg1 mitigation of memory impairment in mice and increase of hippocampal excitability in anaesthetised rats	[69]
Mitigation of anxiety caused by methamphetamine abuse in rats	[70]
Decrease of iNOS synthase and demyelination scores in the central nervous system in mice with experimental autoimmune encephalomyelitis	[71]
Protection against scopolamine-induced memory deficits in rats	[72]
Neuroprotection of pseudoginsenoside F11 on 6-hydroxydopamine-induced Parkinson's disease in rats	[73]
Inhibition of neuronal apoptosis and decrease of neurite damage via regulation of endoplasmic reticulum stress after acute spinal cord injury in rats	[74]
F11 inhibition of hyper locomotion and increase of extracellular dopamine release by GABAergic neurons and μ-opioid receptors regulation in mice	[75]
Modulation of brain function by inhibition of neuronal discharge frequency in brainstem unitary activity in rats	[76]
Neuroprotection on anxiety-like behaviour induced by sleep deprivation in mice	[77]
